# *Dictyota* defense: Developing effective chemical protection against intense fish predation for outplanted massive corals

**DOI:** 10.7717/peerj.14995

**Published:** 2023-03-08

**Authors:** Cailin Harrell, Diego Lirman

**Affiliations:** Department of Marine Biology & Ecology, University of Miami, Miami, Florida, United States

**Keywords:** Fish predation, Coral outplanting, Coral restoration, Novel techniques, Dictyota, Chemical protection, Massive corals, Macroalgae, Florida, Coral survivorship

## Abstract

The incorporation of coral species with massive (*e.g*., boulder, brain) morphologies into reef restoration is critical to sustain biodiversity and increase coral cover on degraded reef ecosystems. However, fragments and colonies of massive corals outplanted in Miami-Dade County, Florida, US, can experience intense predation by fish within the first week of outplanting, resulting in >70% mortality. Here, we tested for the first time the potential benefit of feeding corals powdered *Dictyota*, a brown reef alga that is chemically defended against grazing, to determine if exposure to *Dictyota* can confer chemical protection to coral fragments and reduce the impacts of fish predation after outplanting. We found that feeding corals every 2 to 3 days for 2 months with dried and powdered *Dictyota* prior to outplanting significantly reduced predation levels on *Orbicella faveolata* and *Montastraea cavernosa* fragments (with less than 20% of the fragments experiencing predation up to 1-month post-outplanting). We also found that a single exposure to *Dictyota* at a high concentration 1 to 2 days prior to outplanting significantly reduced predation for six coral species within the first 24 h following outplanting. Thus, feeding corals dry *Dictyota ex situ* prior to outplanting appears to confer protection from fish predation during the critical first days to weeks after outplanting when predation impacts are commonly high. This simple and cheap method can be easily scaled up for corals kept *ex situ* prior to outplanting, resulting in an increase in restoration efficiency for massive corals in areas with high fish predation.

## Introduction

Coral reefs are diverse and highly productive ecosystems that not only support thousands of marine animal and plant species, but also provide a variety of beneficial services for human populations around the globe, such as coastal protection and food provisioning (Beck et al., 2018; Woodhead et al., 2019). Since the 1980’s, total coral cover on Caribbean reefs has been declining at a rate of around 1.4%/year due to climatic and anthropogenic stressors, making total coral cover below 80% for a majority of the region (De’ath et al., 2012; Lester et al., 2020). The negative impacts of a combination of global stressors, such as ocean acidification, increased ocean temperatures, increased hurricane activity, and disease outbreaks have prompted the global expansion of ecological restoration efforts (Ellis et al., 2019; Boström-Einarsson et al., 2020) In Florida, reef restoration efforts have focused primarily on the propagation and outplanting of branching corals from the threatened genus *Acropora*, as these corals experienced massive population declines due to a combination of habitat degradation, disease outbreaks, and thermal stress (Lirman, 2014; van Woesik et al., 2021). Taking advantage of the enhanced growth rates *Acropora* experience following fragmentation, restoration efforts for these species have succeeded in restoring small reef portions (108–300 m^2^ on average) with coral outplant survival at >70% (Schopmeyer et al., 2017; Bayraktarov et al., 2019; Ware et al., 2020; Banister & van Woesik, 2021). However, to reestablish fully functioning coral reef ecosystems, restoration efforts need to incorporate additional important reef-building taxa (Lirman & Schopmeyer, 2016; Lustic et al., 2020).

Atlantic and Caribbean coral species with massive colony morphologies (hereafter “massive corals”), such as those in the genera *Orbicella* and *Pseudodiplora*, provide significant reef complexity and support wider ranges of fish assemblages, which enhance overall ecosystem productivity (Rippe et al., 2017; Syms & Jones, 2000). The incorporation of these species in restoration efforts would fill more functional ecosystem groups necessary to the success of these restored coral communities. In addition, declines in the abundance of massive corals due to the outbreak of Stony Coral Tissue Loss Disease (SCTLD) provide compelling motivation to incorporate these corals into restoration (Brandt et al., 2021; Kolodziej et al., 2021; Meiling et al., 2021). Initially excluded from restoration efforts due to slower growth rates compared to branching coral species, the development of the microfragmentation process, which increases the growth of massive species significantly, has made these taxa more viable for large-scale restoration efforts (Lough & Cantin, 2014; Forsman et al., 2015; Page, Muller & Vaughan, 2018; Costa et al., 2021).

However, massive corals outplanted onto Florida reefs also experience intense predation by parrotfish (Scaridae) and butterflyfish (Chaetodontidae) that impair restoration efforts (Koval et al., 2020; Rivas et al., 2021). Fish predation on massive corals occurs primarily within the first days to weeks after outplanting and decreases in intensity as time progresses (with one study finding that over 50% of its outplanted corals experienced signs of predation after the first week) (Smith et al., 2021). The predation varies across coral species, with one study finding higher predation levels for *Pseudodiploria strigosa* (up to 73%) compared to *Orbicella faveolata* (62%) and *Montastraea cavernosa* (26%) (Koval et al., 2020). These high levels of predation limit the ability of these coral outplants to regenerate damaged tissue, thus impacting the survivorship and growth of these outplanted corals (Meesters, Noordeloos & Bak, 1994). In addition, fish predation can disrupt the microbiome of the predated coral, which may make the coral more susceptible to stressors, like bleaching or disease (Maher et al., 2020). Survivorship and growth of coral outplants are commonly used as indicators of the success of coral restoration efforts. Therefore, the development and implementation of novel outplanting methods that effectively deter fish predation are necessary for the success of coral restoration efforts using massive corals in regions with high fish predation (Lirman et al., 2022).

Physical protection methods have been tested to limit fish predation. These included embedding coral fragments into cement bases so that the edges of the corals were not exposed (Koval et al., 2020), as well as deploying metal spikes and cages around outplanted corals (Rivas et al., 2021). The coral fragments with exposed skeleton had significantly more tissue removed by fish compared to coral fragments embedded into the cement. The spikes and cages were very effective at limiting fish predation, but the protection was lost once these barriers were removed to mitigate cage maintenance, making this approach hard to scale up. Thus, there is still a need to develop efficient protection methods that can be easily expanded and protect corals at the time when they are most susceptible to predation.

Brown macroalgae contain compounds that deter consumption by fish. For example, dipterenes found in the brown macroalga *Dictyota caribaea* protect this species against herbivory (Simas et al., 2014). Similarly, pachydictyol-A produced by *Dictyota*, and certain species of seagrass can significantly reduce consumption by fishes and urchins (Hay & Fenical, 2003; Taylor et al., 2003). We hypothesized here that the secondary metabolites used by the algae to reduce herbivory could potentially be used as chemical protection for corals as documented in the mutualism between monarch butterflies and milkweed plants. Through the consumption of milkweed-containing toxic metabolites, (*i.e*., cardenolides), monarch butterflies experience significant reductions in parasitic infestations compared (Gowler et al., 2015). If metabolites from other organisms can be consumed and incorporated without adverse effects, this could act as a viable chemical defense mechanism for outplanted corals.

The objective of this study was to test novel chemical protection as a method to reduce fish predation on fragments of massive coral species, especially during the first days to weeks after outplanting when the impacts of fish predation are concentrated (Koval et al., 2020; Rivas et al., 2021). We hypothesize that feeding/exposing corals to chemically defended algae *ex situ* prior to outplanting would confer chemical protection against fish predation and mitigate predation impacts, increasing the efficiency of restoration using massive coral species in regions with high fish predation.

## Materials and Methods

### Chronic *Dictyota* feeding

**Figure 1:**
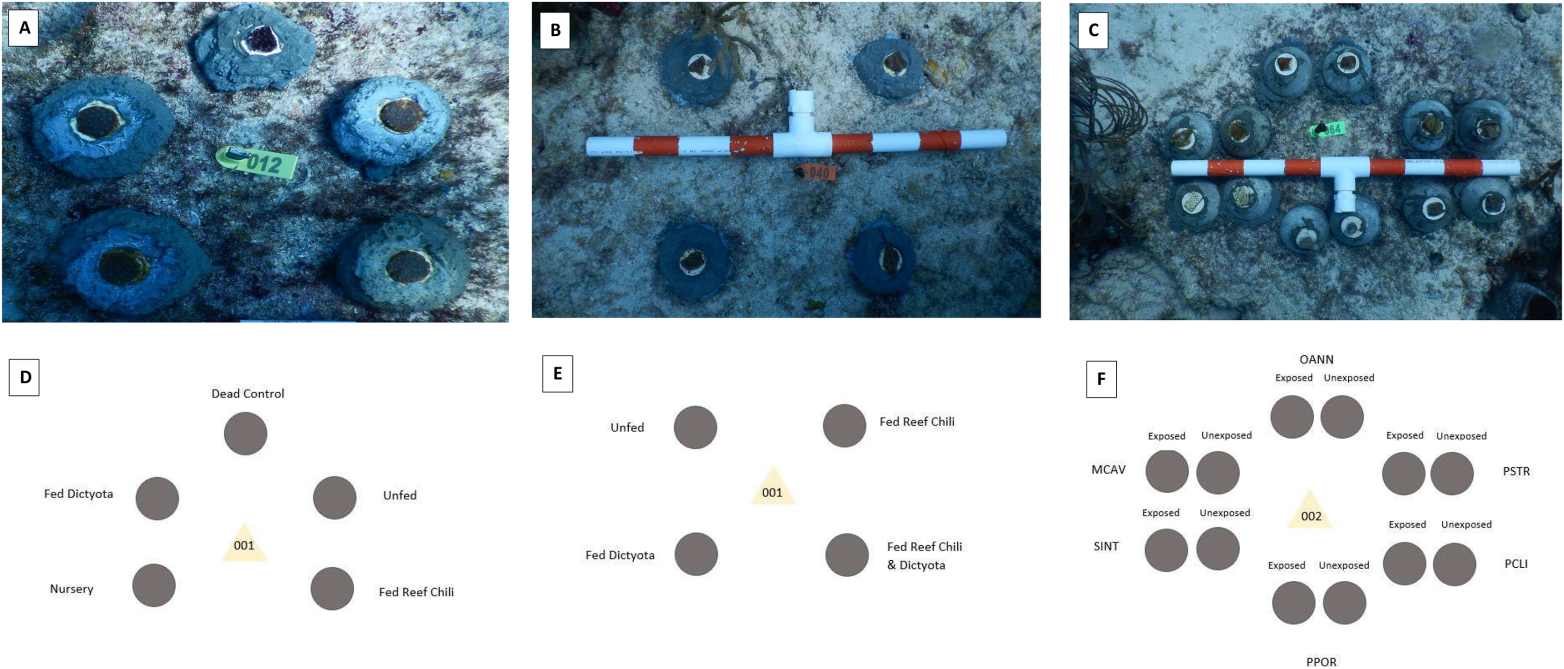
Outplanting designs for each experiment in the study. Configuration of each cluster of outplanted coral fragments. The images were taken after outplanting of the *O. faveolata* (A), *P. strigosa* and *M. cavernosa* (B), and doused coral fragments (C). The plot diagrams depict which treatment went in each position around the tag for the outplanted *O. faveolata* (D), *P. strigosa* and *M. cavernosa* (E), and doused coral fragments (F). The fifth coral treatment (labeled Nursery) in the *O. faveolata* plots (A and D) was used for a separate predation study and therefore not discussed throughout the publication.

Dried and powdered tissue from *Dictyota* spp. was used as a feeding supplement to test chemical deterrence on fish predation. For this experiment, one colony of *Orbicella faveolata* was collected from the Port of Miami (25.776°N, 80.160°W, depth = 2 m) in June 2021. The single colony was fragmented using a band saw to create 120 fragments (3–4 cm in diameter) that were separated into an experimental *Dictyota* treatment (*n* = 20 fragments) and two control treatments (*n* = 45 fragments per treatment). The first control treatment (labeled “fed”) consisted of feeding the *O. faveolata* fragments 50–75 mg of Reef Chili, a common coral feeding supplement, dissolved in approximately 120 mL of seawater. The water flow in the tanks was turned off for the 30-min feeding period which occurred three times a week for 2 months prior to outplanting (Forsman et al., 2012). The second control treatment (labeled “unfed”) was given no supplemental food for the duration of the acclimation period. For the experimental treatment, *Dictyota* was gathered from reef sites off the coast of Miami, dried for 24 h, and pulverized with a mortar and pestle into a powder of similar consistency to Reef Chili. This treatment (labeled “*Dictyota* fed”) consisted of the same methods as the “fed” treatment, with the *Dictyota* powder being used in place of the Reef Chili. The incorporation of *Dictyota* into the coral polyps was confirmed *via* visual observations under a microscope, where fragments were monitored for signs of feeding for the duration of the exposure period (extension of tentacles, closing of coral mouths around the *Dictyota* pieces, *etc*.) (Houlbrèque & Ferrier-Pagès, 2009). An additional dead control treatment was incorporated into the outplanting portion of this study. This treatment consisted of coral skeletons coated with a non-toxic paint (Rit all-purpose dye) to duplicate the coloration of the living fragments. Inclusion of this treatment was made to determine whether parrotfish target outplanted coral fragments for tissue consumption or simply because the outplants are novel items within their territories (Francini-Filho et al., 2008). Fragments of each treatment were outplanted individually at Flamingo Reef (25.70139°N, 80.09817°W, depth = 5–6 m) on September 28, 2021, using cement. The deployment consisted of 45 experimental clusters, each composed of one coral fragment from each treatment (dead control, fed, unfed, *Dictyota* fed) placed 5–10 cm apart from each other (Fig. 1A).

In a second chronic exposure experiment, one colony of *Montastraea cavernosa* and one colony of *Pseudodiploria strigosa*, which had been collected from the Port of Miami (25.776°N, 80.160°W, depth = 2 m) in May of 2021, were fragmented into approximately 120 1-inch square pieces using a diamond bandsaw. The fragments were separated into four treatments (with approximately 30 corals in each treatment): fed only Reef Chili (labeled “fed/RC”), fed only *Dictyota* ((labeled “fed/*Dict*”), 3) fed a combination of Reef Chili and *Dictyota* (labeled fed/RC+D), and not fed any supplements (labeled “unfed”). The feeding regimes for these coral fragments were the same as with the *O. faveolata* fragments, with three feedings a week for 2 months prior to outplanting. Twenty-five fragments from each treatment for both *Pseudodiploria strigosa* and *Montastraea cavernosa* were outplanted at Flamingo Reef (25.701°N, 80.098°W, depth = 5–6 m) in two separate plots on April 27, 2022. The first plot contained *P. strigosa* fragments arranged into 25 clusters (spaced 1 m apart in a grid pattern) with the four conditioning treatments replicated within each cluster (fragments were spaced 5–10 cm apart from each other) (Fig. 1B). The second plot, using the same arrangement and sample size, had *M. cavernosa* fragments from each treatment.

### Acute *Dictyota* exposure

An acute exposure study was conducted with fragments from six coral species (*M. cavernosa*, *P. strigosa*, *Stephanocenia intersepta*, *Porites porites*, *Orbicella annularis*, and *Pseudodiploria clivosa*). One small colony of each species, which had been collected from the Port of Miami in May of 2021, was cut into approximately 20 fragments using a diamond bandsaw. One half of the fragments from each species (*n* ≈ 10) were placed in a separate container with a small pump inside and doused with 25 mL of powdered *Dictyota* (diluted in 300 mL of seawater). These fragments remained in the *Dictyota* “bath” for 3 h 1–2 days prior to outplanting. The remaining half of the fragments of each species were not removed from the original holding aquaria and not exposed to any *Dictyota* prior to outplanting. All the fragments (both exposed and unexposed to *Dictyota*) from all six species were outplanted onto Flamingo Reef in 10 clusters (spaced 1 m apart). Each cluster had a total of 12 fragments, with two fragments of each species, one that had been exposed to *Dictyota* and one that had not. The two fragments of each species were spaced approximately 5 cm from the fragments of the other treatment. The *Dictyota* exposed (fed) and unexposed (unfed) fragments of each species were positioned adjacent to one another (Fig. 1C).

### Predation surveys

Following outplanting, a picture of each coral fragment was taken with an Olympus Tough TG-6 camera attached to a PVC frame to standardize the scale of the images for analysis in ImageJ software. Initial images were taken the day of outplanting and used as a baseline to determine new predation/ bite scars in subsequent surveys, especially between the 1 week and 1 month surveys when algal colonization of bites made new predation less apparent. *Orbicella faveolata*, *M. cavernosa*, and *P. strigosa* fragments were surveyed at 24 h, 1 week, and 1 month post outplanting. Coral condition data (bitten, completely removed, no predation) were recorded by divers at each survey interval (1 day, 1 week, and 1 month). Completely removed and bitten coral fragments were grouped into one category (predation present) for statistical analyses, while the fragments with no predation were grouped into a different category (predation absent). Once an outplant experienced predation (bitten or removed), the coral was not included in the predation present category in subsequent surveys (*i.e*., 1 week or 1 month) if predation occurred again. This strategy allowed for the statistical calculation of the predation probability at each time point separate from previous predation, showing the effectiveness of the treatments over time. Corals from the acute feeding experiment were surveyed using the same methods as the chronic feeding experiments at 24 h, 1 week, and 1 month after outplanting.

### Lipid and protein content analysis

Tissue samples (*n* = 5) were taken from the *O. faveolata* colony used in the chronic feeding experiment prior to conditioning as well as from each of the treatments (fed, unfed, *Dictyota* fed) at the conclusion of the conditioning period (*i.e*., prior to outplanting). Lipid content of all the samples (initial and final) was measured *via* solvent extraction and gravimetric analysis of the filtered blastate (Teece et al., 2011). Protein content was obtained with a Pierce BCA protein assay kit, using the microplate procedure (Thermo Fisher Scientific, 2019).

### Statistical analyses

All analyses were conducted in R Programming Language version 4.0.2 (RStudio Team, 2020). We utilized a Generalized Linear Model (GLM) with a binomial distribution and logit link function to evaluate any differences in the probability of predation of predation on the outplanted corals, as described by Warton & Hui (2011) and utilized in other research regarding fish predation on massive corals (Koval et al., 2020; Rivas et al., 2021). The predation status (present or absent) was our binary response variable while treatment type and survey time point were the fixed effects predictor variables used in the model. The model with the best fit quantified the probability of predation based on the interaction between treatment type and survey time point (predation ~ treatment + survey time point) for the chronic feeding experiments. For the acute feeding experiment, species was included as another fixed effect predictor variable in the model (predation ~ species + treatment + survey time point). Tukey *post hoc* tests were then used to evaluate the significant differences among the levels of the model’s categorical variables (treatment, survey time, and species for the acute experiment). The total percentage of outplants in each treatment that experienced predation was calculated by summing up the total numbers of newly bitten or removed outplants at each time point and dividing by the total number of outplants in that treatment. The average lipid and protein contents for the initial tissue samples of *O. faveolata* were compared to the post-conditioning tissue samples with an ANOVA and Tukey *post hoc* test to evaluate any pairwise differences between them (Rivas et al., 2021).

## Results

### Chronic *Dictyota* feeding

Microscopic observations revealed the active consumption of the powdered *Dictyota* by corals during feeding trials. These observations also revealed particles of *Dictyota* powder getting trapped in the coral mucus layer, which would act as another form of *Dictyota* retention in the corals. No coral mortality was observed during the *Dictyota* feeding trials, indicating that the consumption of *Dictyota* does not directly cause coral tissue mortality. Less than 2% of the outplanted corals in the dead control treatment experienced fish predation (with that predation occurring within the first 24 h following deployment). Given the minimal amount of predation compared to the other treatments, the dead control corals were excluded from further analyses.

**Figure 2:**
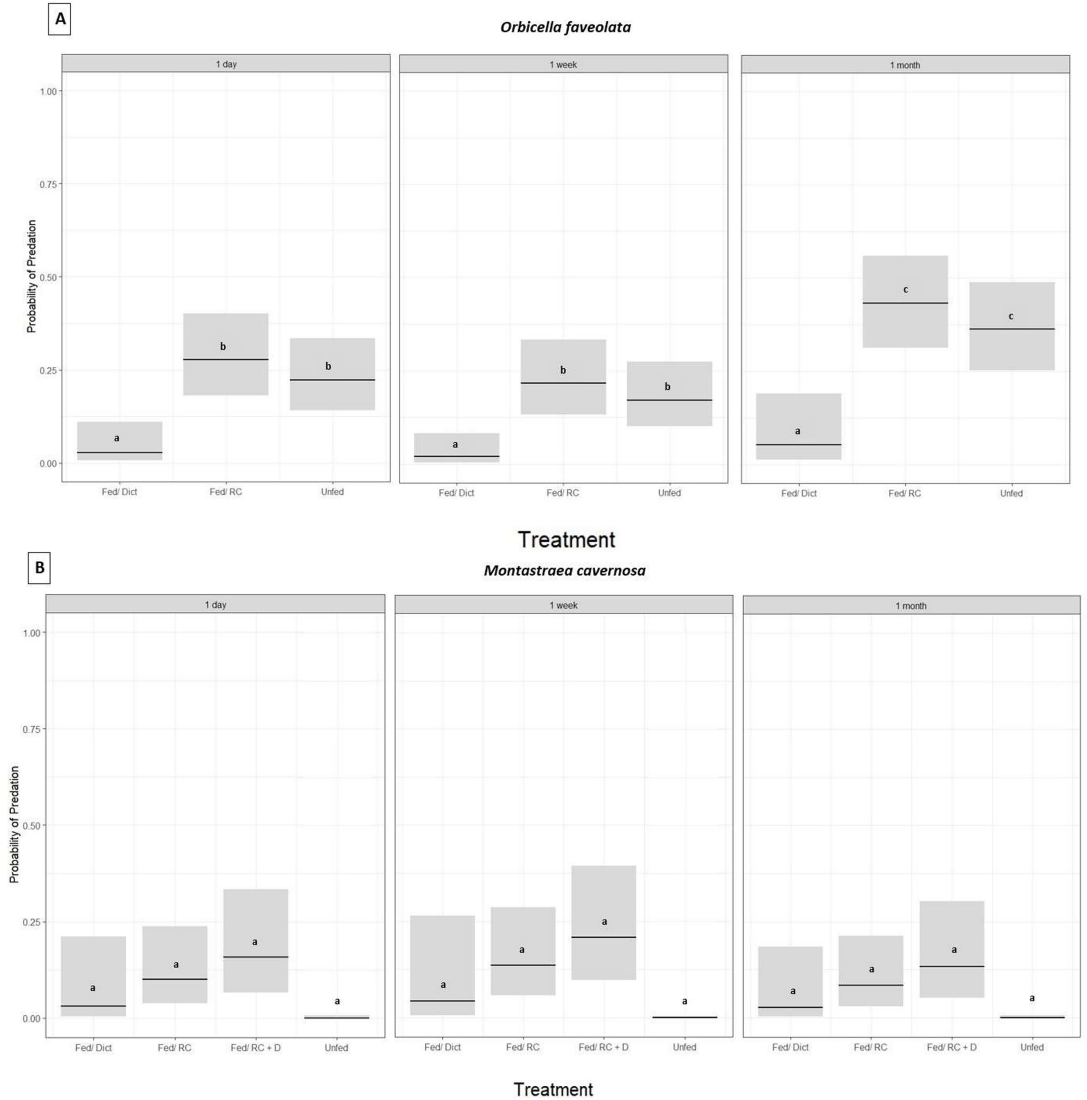
Predation probability results for *Orbicella faveolata* and *Montastraea cavernosa* fragments chronically fed *Dictyota* algae prior to outplanting. Probability of predation (presence of fish bites on or total removal of coral fragments) based on treatment administered prior to outplanting and grouped by survey time, estimated using a binomial GLM for *O. faveolata* (A) and *M. cavernosa* (B). Bars indicate GLM fitted values (center lines) and 95% confidence intervals. Tukey HSD pairwise test comparisons: *O. faveolata* (A): Fed/Dict (*Dictyota*) ≠ Fed/RC (Reef Chili) = Unfed (Not fed) and for *M. cavernosa* (B): Fed/Dict (*Dictyota*) = Fed/RC (Reef Chili) = Unfed (Not fed) = Fed/ RC + D (Fed combination of Reef Chili & *Dictyota*).

*Orbicella faveolata* fragments fed *Dictyota* had significantly lower probability of predation compared to the corals in the fed/Reef Chili and unfed treatments (GLM,*p* ≤ 0.05, Tukey HSD, *p* ≤ 0.05 for all treatments) (Fig. 2A) (Table 1). In addition, there was no statistically significant difference in the probability of predation between the fed/Reef Chili and unfed treatments (Tukey HSD, *p* = 0.88) (Fig. 2A). There was also a statistically significant difference in predation probability among the three survey time points (GLM, *p* ≤ 0.05), with predation probability at 1 month being significantly higher than the earlier surveys (1 day and 1 week) (Tukey HSD, *p* ≤ 0.05). At the end of 1 month, 18% of the outplanted *Orbicella* fragments in the *Dictyota* treatment had experienced predation, while the fed/Reef Chili and unfed treatments experienced predation on 90% and 73% of their fragments respectively.

**Table 1:** Tukey HSD pairwise test results for the corals chronically fed *Dictyota* (*O. faveolata*, *M. cavernosa*, and *P. strigosa*) prior to outplanting.

	Interaction	*p* value
*O. faveolata*		
Treatment		
	Fed/Reef Chili-Fed/Dictyota	0.000008
	Unfed-Fed/Dictyota	0.0002
	Unfed-Fed/Reef Chili	0.886
Time		
	1 month-1 day	0.075
	1 week-1 day	0.676
	1 week-1 month	0.007
*P. strigosa*		
Treatment		
	Fed/Reef Chili-Fed/Dictyota	0.1540
	Fed Mix-Fed/Dictyota	0.7174
	Unfed-Fed/Dictyota	0.9078
	Fed Mix-Fed/Reef Chili	0.7109
	Unfed-Fed/Reef Chili	0.5813
	Unfed-Fed Mix	0.9907
Time		
	1 week-1 day	0
*M. cavernosa*		
Treatment		
	Fed/Reef Chili-Fed/Dictyota	0.666
	Fed Mix-Fed/Dictyota	0.192
	Unfed-Fed/Dictyota	0.976
	Fed Mix-Fed/Reef Chili	0.651
	Unfed-Fed/Reef Chili	0.424
	Unfed-Fed Mix	0.098
Time		
	1 week-1 day	0.5687

**Note:**

The interactions describe which variables (species, treatment, or time survey point) had significantly different influences over the predation probability, with *p*-values < 0.05 indicating a significant difference between variables.

For *Pseudodiploria strigosa*, there was no significant difference in the probability of predation among the four treatments (fed/Reef Chili, fed/*Dictyota*, fed/Reef Chili + *Dictyota*, or unfed) after 1 day (GLM, *p* = 0.12). At 1 week post outplanting, every single *Pseudodiploria* fragment in all four treatments was completely removed or experienced over 95% tissue loss to predation. *Montastraea cavernosa* fragments also showed no significant difference in the probability of predation among the four treatments (fed/Reef Chili, fed/*Dictyota*, fed/Reef Chili + *Dictyota*, or unfed) (GLM, *p* = 0.09) (Fig. 2B) (Table 1). There was no significant difference in predation probability among the three survey time points for *M. cavernosa* (GLM, *p* = 0.67). At the end of 1 month, 10% of the outplanted *Montastraea* fragments in the *Dictyota* treatment had experienced predation, while the fed/Reef Chili, fed/Reef Chili + *Dictyota*, and unfed treatments experienced predation on 32%, 40%, and 0% of their fragments respectively. The outplanted *Pseudodiploria* fragments experienced 100% predation across all treatments by the end of the survey period.

### Acute *Dictyota* exposure

**Figure 3:**
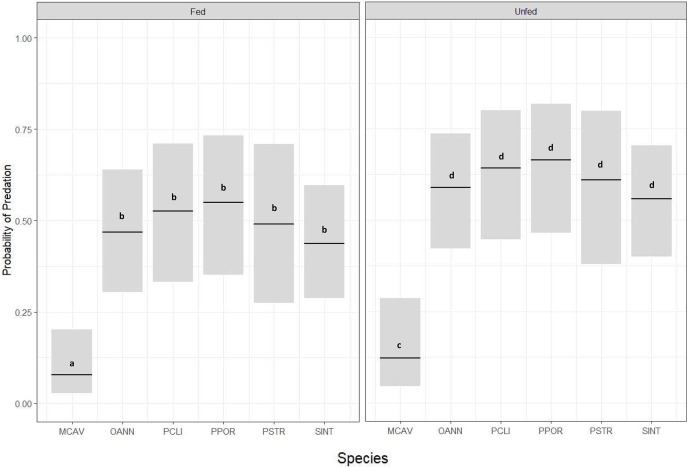
Predation probability results for coral fragments doused with *Dictyota* algae in the days prior to outplanting. Probability of predation (presence of fish bites on or total removal of coral fragments) at 24-h survey based on species and grouped by treatment (Fed or Unfed), estimated using a binomial GLM for MCAV (*M. cavernosa*), OANN (*O. annularis*), PCLI (*P. clivosa*), PPOR (*P. porites*), PSTR (*P. strigosa*), and SINT (*S. intersepta*). Bars indicate GLM fitted values (center lines) and 95% confidence intervals. Tukey HSD pairwise test comparisons: Fed ≠ Unfed; MCAV ≠ OANN = PCLI = PPOR = PSTR = SINT.

Corals from all six species had significantly lower predation probability when exposed to *Dictyota* (fed) compared to those that were not exposed to *Dictyota* (unfed) prior to outplanting (GLM, *p* = 0.05) (Fig. 3) (Table 2). There was also a significant difference in predation probability among species, with *M. cavernosa*, in both the exposed and unexposed treatments, having significantly less predation compared to the other five species (Tukey HSD, *p* ≤ 0.05) (Fig. 3). Except for *M. cavernosa*, the other five outplanted species suffered total removal of fragments between the 24-h survey and the 1 week survey (similar to that of the *P. strigosa* outplanted in the chronic *Dictyota* feeding trial). As a result, predation probability was significantly higher at 1 week compared to 1 day post outplanting (Tukey HSD, *p* ≤ 0.05).

**Table 2:** Tukey HSD pairwise test results for the corals doused with *Dictyota* prior to outplanting.

	Interaction	*p* value
Species		
	OANN-MCAV	0.0000075
	PCLI-MCAV	0.00001
	PPOR-MCAV	0.0000015
	PSTR-MCAV	0.0002
	SINT-MCAV	0.005
	PPOR-OANN	0.99
	PSTR-OANN	0.99
	PCLI-OANN	0.99
	SINT-OANN	0.42
	PPOR-PCLI	0.99
	PSTR-PCLI	0.99
	SINT-PCLI	0.38
	PPOR-PSTR	0.98
	PPOR-SINT	0.16
	PSTR-SINT	0.66
Treatment		
	Unexposed-Exposed	0.009

**Note:**

The interactions describe which variables (species, treatment, or time survey point) had significantly different influences over the predation probability, with p-values < 0.05 indicating a significant difference between variables.

### Lipid and protein content

There was no significant difference in the average lipid content of *O. faveolata* tissue samples taken from the colony prior to conditioning (2,828.6 mg/cm^2^) and the tissue samples taken from each treatment following conditioning: fed (2,233.6 mg/cm^2^), unfed (2,725.9 mg/cm^2^), and *Dictyota* fed (2,247.6 mg/cm^2^) (Tukey HSD, *p* > 0.05). In addition, there was no significant difference in average protein concentration (ug/mL) between the pre- and post-conditioning tissue samples (ANOVA, *p* = 0.97).

## Discussion

Predation by fish (parrotfishes and butterflyfishes) has emerged as a recent restoration bottleneck in regions like Miami, Florida, where coral outplants of massive taxa can experience rapid and significant losses (Koval et al., 2020; Rivas et al., 2021; Lirman et al., 2022). In this study, we tested for the first time the potential to chemically defend outplanted massive coral fragments against fish predation, with the marine macroalgae *Dictyota* as the source of chemical protection. We found that feeding powdered *Dictyota* to *O. faveolata* for 2 months prior to outplanting significantly reduced the probability of those fragments being consumed by corallivorous fish up to 1 month post outplanting. However, other species of massive corals, *P. strigosa* and *M. cavernosa*, did not significantly benefit from being fed *Dictyota* prior to deployment. We also found that only exposing massive coral fragments to *Dictyota* algae shortly before outplanting (within 48 h of deployment) does not provide significant protection against predation beyond 24 h, regardless of the species used.

Members of the brown algal genus *Dictyota* contain secondary metabolites within their tissues that have been found to deter grazing from a variety of herbivorous taxa (Chen et al., 2018). In both marine and terrestrial ecosystems, chemically defended organisms are utilized as a defense mechanism by others. For example, monarch butterflies consume and retain the secondary metabolites found within milkweed to deter predation (Baker & Potter, 2018). This also occurs in ecosystems where symbiotic microorganisms can confer chemical protection to their hosts. The larvae of *Bugula neritina*, a marine bryozoan, uptake a bacterium, *Candidatus Endobugula sertula*, that effectively deters predation from the pinfish *Lagodon rhomboides* (Lopanik, 2014). Given the existence of these ecological relationships, we expected the secondary metabolites within *Dictyota* to be utilized as a chemical defense following consumption by coral fragments. *Orbicella faveolata* fragments fed *Dictyota* had 11%, 15%, and 80% less predation compared to the corals fed Reef Chili at 1 day, 1 week, and 1 month respectively. We have found that feeding *Orbicella* fragments *Dictyota* algae for 2 months prior to outplanting can reduce corallivorous fish predation, supporting the potential development of the hypothesized ecological relationship mentioned previously.

While we visually confirmed pieces of *Dictyota* entering the mouths of the corals during feeding, this does not guarantee that the corals digested and incorporated the secondary metabolites found in *Dictyota* for protection against predation. Due to a limitation of resources, we were unable to conduct assays and confirm that the *Dictyota* secondary metabolites, such as pachydictol A, were incorporated into the tissues of the coral fragments following feeding. The use of coral metabolomics or isotope tracing can reveal the chemical makeup of the coral holobiont pre and post *Dictyota* feeding, which may provide details on whether or not the secondary metabolites were actually within the coral tissue following consumption (Hemsing et al., 2018; Greene et al., 2020). These types of analyses would be necessary to confirm that our results in this study were due to the uptake and retention of *Dictyota* by the corals and not another physical or environmental variable.

If the *Dictyota* was not actively used by the corals following consumption, there is still a potential that the corals were chemically defended against fish predation another way. In addition to active consumption, the *Dictyota* particles, and therefore secondary metabolites, could have been trapped within the coral’s surface mucus layer (SML) upon exposure. The SML is a gel-like substance produced by cells in the coral’s epithelium that acts as a protective barrier for the coral’s tissue and as a heterotrophic feeding mechanism for suspended particles in the marine environment (Brown & Bythell, 2005; Wooldridge, 2009; Bythell & Wild, 2011)(Brown & Bythell, 2005; Wooldridge, 2009; Bythell & Wild, 2011). To test whether the trapping of *Dictyota* in the coral SML would be a sufficient means of chemical protection, we exposed fragments from six massive coral species (*M. cavernosa*, *P. strigosa*, *Stephanocenia intersepta*, *Porites porites*, *Orbicella annularis*, and *Pseudodiploria clivosa*) to a highly concentrated dose of *Dictyota* powder in the 2 days prior to deployment. We saw that this acute *Dictyota* exposure was effective 1 day post outplanting, as the fragments in the *Dictyota* exposed treatment had significantly less predation compared to the unexposed treatment across all six species. However, this protection did not last, as the outplanted fragments in all six species experienced significantly increased predation at the 1 week survey. While these results indicate that acute exposure would not be an effective method to reduce fish predation on massive coral outplants, they are not unexpected. The coral mucus layer is usually shed to clean sediments off the surface of the corals or protect the corals from environmental stressors (Brown & Bythell, 2005; Mayer et al., 2010). So, the disappearance of any predation protection provided by the *Dictyota’s* secondary metabolites after 24 h was probably the result of natural mucus sloughing and cannot be avoided in attempts to prevent predation *via* acute chemical exposure in the future.

While *O. faveolata* fragments exhibited lower levels of predation after being exposed to *Dictyota*, the same cannot be said for either *M. cavernosa* or *P. strigosa. Montastraea cavernosa* fragments showed no significant difference in the probability of predation among the four treatments. The *P. strigosa* fragments experienced extreme predation 1 week after outplanting, with 100% of the fragments being completely removed or consumed. Thus, much like how predation impacts appear to vary among coral species, as seen in Koval et al.’s (2020) research, *Dictyota* protection against fish predation also appeared to vary among coral species in the chronic feeding trials. In this study, we were only able to use one genotype of each coral species due to the limited availability of these coral species for use in an experimental trial. As a result, the variation in predation seen among species may only be because the single genotype used may have been extremely palatable or unpalatable to the parrotfish, which has been seen before in the predation study conducted by Rivas et al. (2021). Individual coral genotypes can vary greatly in their metabolic makeup, which can impact the coral’s susceptibility to environmental stressors like disease or predation (Lohr et al., 2019). Since we only used one genotype per species of coral in this study, we cannot assume that some coral species are more protected from predation by *Dictyota* exposure. To confirm that certain coral species, like *O. faveolata*, experience reduced fish predation when exposed to *Dictyota* prior to outplanting, future experiments should incorporate multiple genotypes of each species used, if possible.

Along with the influence of using singular coral genotypes, the predation levels seen among species could have been influenced by the dead control treatment, which was only present in the *Orbicella* outplanting. The dye used to make the coral skeletons simulate living coral tissue leached from the skeletons over time, as evidenced by the complete loss of pigmentation in these corals by the 1 month surveys. Although the dye was labeled as non-toxic, there may have been chemicals present that deterred fish from the plot area. Fish can utilize chemical cues to determine the risk, usually predation associated, of an area in the marine environment. The presence of unfamiliar chemicals, like those present in the dye we utilized, could prompt the fish to avoid that area because there may be danger present (Brown & Chivers, 2006; Wisenden, 2008). This would provide another explanation as to why predation was much higher in the *Pseudodiploria* and *Montastraea* outplants when compared to the *Orbicella* outplants because there were no dyed, dead controls used in the *Pseudodiploria* or *Montastraea* plots. Exploring how these limitations impacted the effectiveness of *Dictyota* feeding would be important in determining the applicability of these results. If our results are solely the effect of these limitations (single genotype resistance or toxic chemical presence), the methodology would not be as effective in mitigating predation for massive coral restoration efforts as previously described.

Since *Dictyota* feeding may be a potentially effective predation mitigation tactic for massive coral species, it is important to consider the potential trade-offs of adding *Dictyota* to a coral’s diet during *ex-situ* culture prior to outplanting. A primary concern of ours with using *Dictyota* was that the ingestion of the predation deterring secondary metabolites within the macroalgae’s tissue would cause mortalities among the coral fragments prior to outplanting, as several studies have found that allelopathic compounds in some macroalgae species can directly harm corals (Rasher & Hay, 2010; Rasher et al., 2011). We did not observe any fragment mortalities during the chronic feeding or acute exposure trials, indicating that the ingestion of this algae does not cause immediate harm to the corals. However, we were unable to conduct assays of the coral microbiome and metabolome following the feeding of *Dictyota* to the corals. Therefore, we have no insight on potential negative impacts the chemicals found in *Dictyota* could have on the corals, like reduced rates of photosynthesis, respiration, or growth which have been seen in other chemical/toxin exposure studies on corals and coral larvae (Reichelt-Brushett & Harrison, 2004). In addition, the longest feeding period (2 months) may have been too short to reveal any delated or long-term negative impacts on corals that could occur following prolonged periods of *Dictyota* ingestion. To fully determine the safety of this algae as a coral food supplement aimed at decreasing fish predation, further studies should conduct these types of assays to reveal the full scope of impacts (positive or negative) that *Dictyota* in feeding can have on a coral fragment. Once these impacts have been noted, the regular inclusion of *Dictyota* in feeding regimes for massive coral fragments meant for outplanting on high predation-risk reefs could be considered.

Another potential tradeoff presented using *Dictyota* as a feeding supplement is that it may lack nutritional elements that the corals need for optimum growth and survivorship. Coral feeding supplements, like the Reef Chili used in our control feeding treatment, are made up of a variety of components such as zooplankton, phytoplankton, and blue green algae. These components provide the corals with fatty acids, proteins, antioxidants, vitamins, and minerals (Reef Chili 100% Complete Coral Food, https://reefchili.com/index.html). As a macroalgae, *Dictyota* may not be able to provide comparable amounts of these essential nutrients to the corals, although brown algae species have been found to contain high concentrations of proteins and fatty acids (Wielgosz-Collin, Kendel & Couzinet-Mossion, 2016; Chavan & Yadav, 2022). However, we analyzed the lipid and protein contents of *O. faveolata* fragments fed *Dictyota* and Reef Chili, finding no significant differences in these nutritional metrics between the two treatments. This indicates that feeding *Dictyota* to coral fragments in place of other traditional coral food supplements may not significantly alter the nutritional health of the corals. Further research into how the lack of specific nutritional compounds provided by *Dictyota* impacts the overall survivorship and growth of corals would be necessary to determine the feasibility of using only *Dictyota* as a food source to limit predation.

## Conclusions

This study expands existing research into one of the major hurdles coral restoration practitioners experience in regions like Miami, Florida, USA: intense fish predation on recently outplanted massive corals. These coral species play critical roles in coral reef ecosystem structure and function. In combination with the population losses brought about by the outbreak of Stony Coral Tissue Loss Disease, the impacts of fish predation on outplanted massive corals have hindered restoration efforts necessary to restore multiple populations of these coral species on Florida’s Coral Reef. The findings of this study provided a potential technique for mitigating fish predation in ways that may make massive coral restoration more feasible. *Dictyota* food supplements served as an effective predation mitigation strategy for *Orbicella faveolata* and could promote higher success rates for restoration efforts targeting this species in this region of Florida’s Coral Reef. Additional research developing predation mitigation tactics that are effective across multiple species of massive coral would be necessary to scale restoration efforts up to the levels of success seen with branching coral species like *Acropora cervicornis*.

## Supplemental Information

10.7717/peerj.14995/supp-1 Supplemental Information 1 R code used to analyze the chronic feeding trial data. This code runs a linear regression to find the best fit model of predation probability based on treatment type and survey time point for each of the three species used in the analysis.

10.7717/peerj.14995/supp-2 Supplemental Information 2 R code used to analyze the acute feeding trial data. The code runs a linear regression of the best fit model of the predation probability for all six coral species used based on treatment type, coral species, and survey time point.

10.7717/peerj.14995/supp-3 Supplemental Information 3 Predation data from the acute feeding trial. The primary data, labeled prevalence, notes the presence or absence of fish bites on the corals by species, treatment type, and survey time point. Presence of predation is indicated by a 1 and absence by a 0. Survey time point indicates the amount of time following outplanting when predation was noted.

10.7717/peerj.14995/supp-4 Supplemental Information 4 Zip archive of relevant data files for *Dictyota* analysis code. The primary data, labeled prevalence, notes the presence or absence of fish bites on the corals by treatment type, and survey time point. Presence of predation is indicated by a 1 and absence by a 0. Survey time point indicates the amount of time following outplanting when predation was noted. Each file in this zip file represents the data for one of the species used in the chronic feeding study, with 3 species used in total.

10.7717/peerj.14995/supp-5 Supplemental Information 5 *Orbicella faveolata* tissue data and analysis for final lipid content. These data represent the final lipid content of the coral tissue samples among the different treatment types. Lipid content was calculated from the resulting values of the filtration process using the equations provided by ThermoScientific.

10.7717/peerj.14995/supp-6 Supplemental Information 6 *Orbicella faveolata* tissue data and analysis for total protein concentration. These data represent the protein concentrations of the coral tissue samples among the different treatment types. Protein concentration was calculated from the resulting values of the protein assay using the equations provided by ThermoScientific.

## Additional Information and Declarations

### Field Study Permissions

The following information was supplied relating to field study approvals (*i.e*., approving body and any reference numbers): Florida Fish and Wildlife Conservation Commission.

### Data Availability

The following information was supplied regarding data availability: The raw data are available in the Supplemental Files. This raw data was analyzed using the R code found in Supplemental Files labeled Dictyota analysis and Douse analysis.
